# Breathlessness and the brain: the role of expectation

**DOI:** 10.1097/SPC.0000000000000441

**Published:** 2019-07-01

**Authors:** Lucy L. Marlow, Olivia K. Faull, Sarah L. Finnegan, Kyle T.S. Pattinson

**Affiliations:** a Nuffield Department of Clinical Neurosciences, University of Oxford, Oxford, UK; b Translational Neuromodeling Unit, Institute for Biomedical Engineering, University of Zurich and ETH Zurich, Zurich, Switzerland

**Keywords:** brain, breathlessness, dyspnoea, expectation, interoception

## Abstract

**Purpose of review:**

Breathlessness debilitates countless people with a wide range of common diseases. For some people, the experience of breathlessness is poorly explained by the findings of medical tests. This disparity complicates diagnostic and treatment options and means that disease-modifying treatments do not always have the expected effect upon symptoms. These observations suggest that brain processing of respiratory perceptions may be somewhat independent of disease processes. This may help to explain the dissonance observed in some patients between physical disease markers and the lived experience of breathlessness.

**Recent findings:**

A body of breathlessness research using functional neuroimaging has identified a relatively consistent set of brain areas that are associated with breathlessness. These areas include the insula, cingulate and sensory cortices, the amygdala and the periaqueductal gray matter. We interpret these findings in the context of new theories of perception that emphasize the importance of distributed brain networks. Within this framework, these perceptual networks function by checking an internal model (a set of expectations) against peripheral sensory inputs, instead of the brain acting as a passive signal transducer. Furthermore, other factors beyond the physiology of breathlessness can influence the system.

**Summary:**

A person's expectations and mood are major contributors to the function of the brain networks that generate perceptions of breathlessness. Breathlessness, therefore, arises from inferences made by the brain's integration of both expectations and sensory inputs. By better understanding individual differences across these contributing perceptual factors, we will be better poised to develop targeted and individualized treatments for breathlessness that could complement disease-modifying therapies.

## INTRODUCTION

Physicians often point out a puzzling phenomenon that one can see two patients with the same lung function (measured objectively) whereby one patient is active, goes out every day, and does many things healthy people do such as work and raise children, whereas the other patient, with the same measured lung function, is severely disabled, housebound, and does very little independently. Havi Carel (2018) [1^▪▪^]

It is well recognized that the subjective experience of breathlessness is often poorly explained by objective disease markers, such as tests of lung [2] and heart function [3]. This discordance extends into the way different people respond to treatment, and can even manifest as unexpected breathlessness, particularly in response to stressful situations. This is illustrated by the following examples:

(1) Pulmonary rehabilitation, a course of exercise and education for people with breathing difficulties, profoundly improves breathlessness yet has no measurable effect on lung function [4–6]. The beneficial effect on breathlessness-related fear and anxiety is noteworthy [7,8].
(2) The monoclonal antibody Mepolizumab has revolutionized the treatment of eosinophilic asthma. However, around 40% of people who are successfully treated (defined by resolution of inflammatory markers) remain symptomatic [9].
(3) Anecdotally, individuals with asthma often report feeling breathless immediately on noticing the loss of their inhaler.
(4) Equally, individuals with lung disease who use ambulatory oxygen report similar sensations and concerns when operating without their cylinder or without a spare cylinder on hand; despite oxygen saturations remaining constant, they can experience significant breathlessness. Symptoms appear ’out of the blue’ in response to the psychological stressor of having the oxygen cylinder, or its spare, out of reach.

An extensive body of literature has provided understanding of the scope of pathophysiological mechanisms that can lead to breathlessness. The importance of lung mechanics, gas exchange, skeletal muscle, diaphragmatic and cardiac function [10,11^▪^,^12^] are well documented. However, it is increasingly clear that peripheral pathophysiology only tells us part of the story, and that brain mechanisms associated with affective state and expectations play an important role. For example, in pain, manipulating expectations can influence subjective perception reports by approximately 30% [13,14^▪▪^]. Therefore, we need to consider the brain as an active participant in the generation of perceptions, so that we can begin to understand the fundamental neural mechanisms by which breathlessness arises.

To date, functional neuroimaging studies have identified a relatively consistent set of brain regions that are active during breathlessness [15–18]. Although this enables more focused hypothesis generation, it must be noted that most studies have not dissociated brain activity that is specific to the sensation of breathlessness (e.g. that scales with self-report) from other concurrent processes that might happen at the same time (e.g. increased work of breathing [19]). As such, it appears that searching for a linear scaling of breathlessness (within and between individuals) with localized activity in a specific ‘breathlessness brain region’ is somewhat unlikely to yield fruitful results. This is primarily because the fundamental actions of the brain are thought to result from distributed network activity, rather than different brain areas having specific functions (e.g. phrenology). Additionally, we know that breathlessness can vary across moments in time, specific episodes and individuals [20]. Thus, instead of focusing on specific breathlessness brain regions, we need to broaden our perspective to investigate the dynamic and distributed brain networks that generate breathlessness, allowing for multiple influencing factors and individual differences. This will help guide understanding beyond where in the brain breathlessness is processed, to how it is processed.

This review explains current evidence on how neural signals from the body are integrated by the brain, leading to perceptions such as breathlessness. We explain how aberrant perceptual processing may help us better understand the disconnect between a person's experience of breathlessness and objective disease markers measured by physicians [1^▪▪^,^21^^▪▪^]. We will then explain how this approach will help develop new treatments for breathlessness. 

**Box 1:**
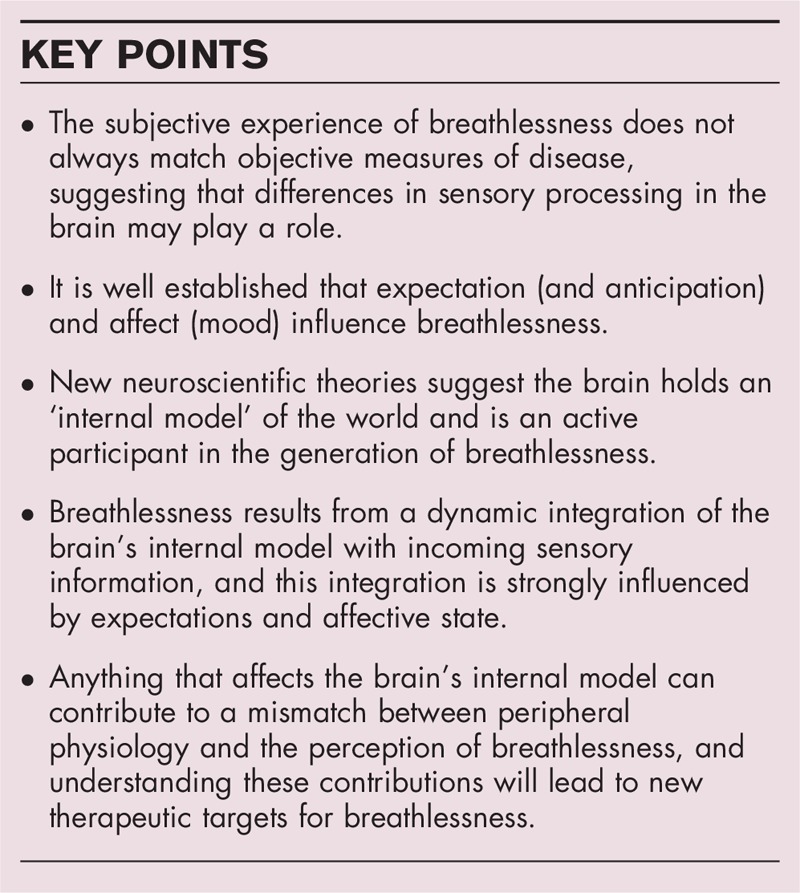
no caption available

## THE PREDICTIVE BRAIN AND INTEROCEPTION

**FIGURE 1:**
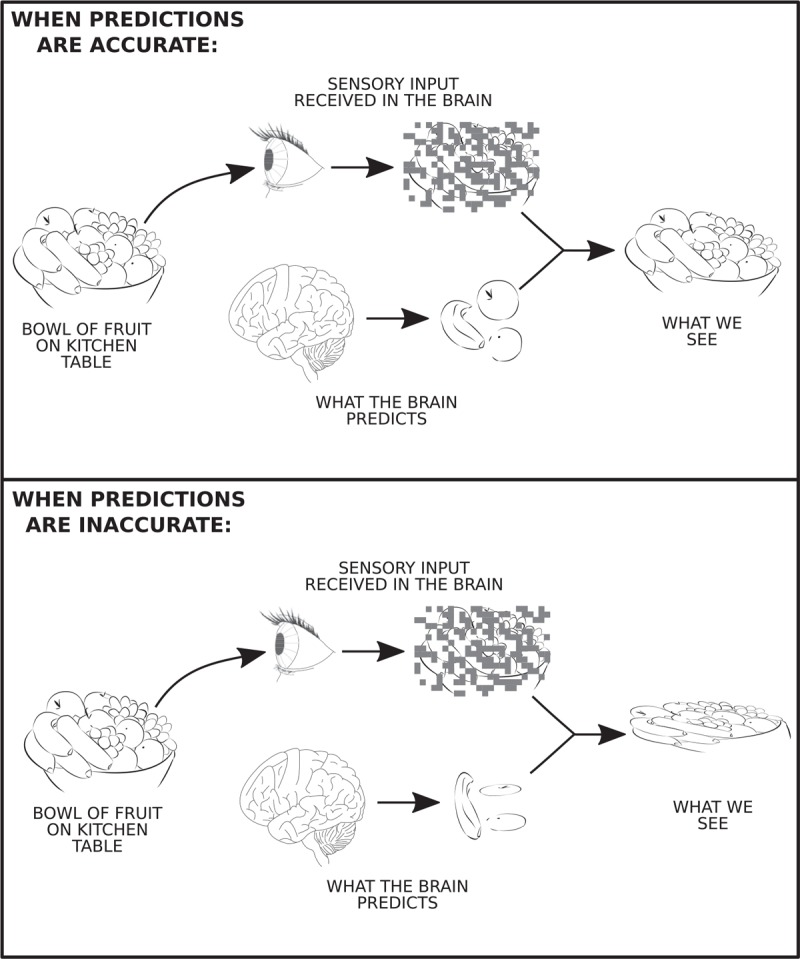
Integration of prediction and sensory input. In order to generate a perception (whether it be visual as in this example or respiratory awareness in the case of breathlessness) the brain has to interpret a noisy set of sensory inputs. To do this we now think that the brain depends upon a set of predictions built out of past experiences and beliefs. The final perception, be it visual or breathing, is thus an inference. Anything that changes the inference may change the perception irrespective of sensory input. When predictions are accurate, what we ultimately see, or feel, is closely related to what is actually present in the environment or our bodies. However, when predictions are inaccurate, our perceptions can be different to the real world (represented here by the distorted bowl of fruit). When predictions about breathing based on past experiences of breathlessness are negative (e.g. sense of failure for not achieving an activity, feeling of social pressure to walk at equal speed to a companion, fear of needing to sit down and not being near a chair), the final perception of breathlessness may be severe, despite the physiological changes not being so significant (a distorted perception).

The brain, encased within the skull, has no direct access to any stimulus, either internal or external to the body. Each person's ‘reality’ is based upon a limited set of (noisy) incoming sensory signals. To decipher these signals, the brain refers to past experiences and beliefs to predict what is happening [22–24]. An example from the visual system is illustrated in Fig. 1, where your brain must make inferences about the missing pixels in the sensory signal. These ideas have more recently been extended beyond the visual system to internal sensations, including breathing [25].

Interoception is a neuroscientific term that encompasses the brain's sensing of stimuli from within the body, both conscious and subconscious [25]. Whilst traditional models of perception considered the brain as a passive stimulus–response organ, current theories promote the brain to play an active role in perception. From this perspective, the brain holds an existing mental model of the world (both external and internal to the body), which is constantly altered and updated via its interaction with incoming sensory inputs [26]. This approach enables predictions about the world's current state to be fed forward through a neural network, allowing model updates via the mismatch between predictions and sensory signals. Prediction error is the neuroscientific term used to describe this mismatch (or error) between what the brain expects (predictions) and what the brain receives (sensory signals). The ideas of predictions and prediction errors have been successfully adopted in simple models that explain learning behaviours, such as those by Rescorla and Wagner [27]. More intricately, Bayesian theories hypothesize that the brain utilizes internal probabilistic models to manage uncertainty amongst the sensory noise [22]. In this framework, the brain deals with uncertainty by generating a probable set of predictions that are informed by past experiences (e.g. struggling to breathe), which are combined with contextual cues from the environment (e.g. fresh flowers that signal pollen and asthma attacks) to regularize the noisy sensory signals.

**FIGURE 2:**
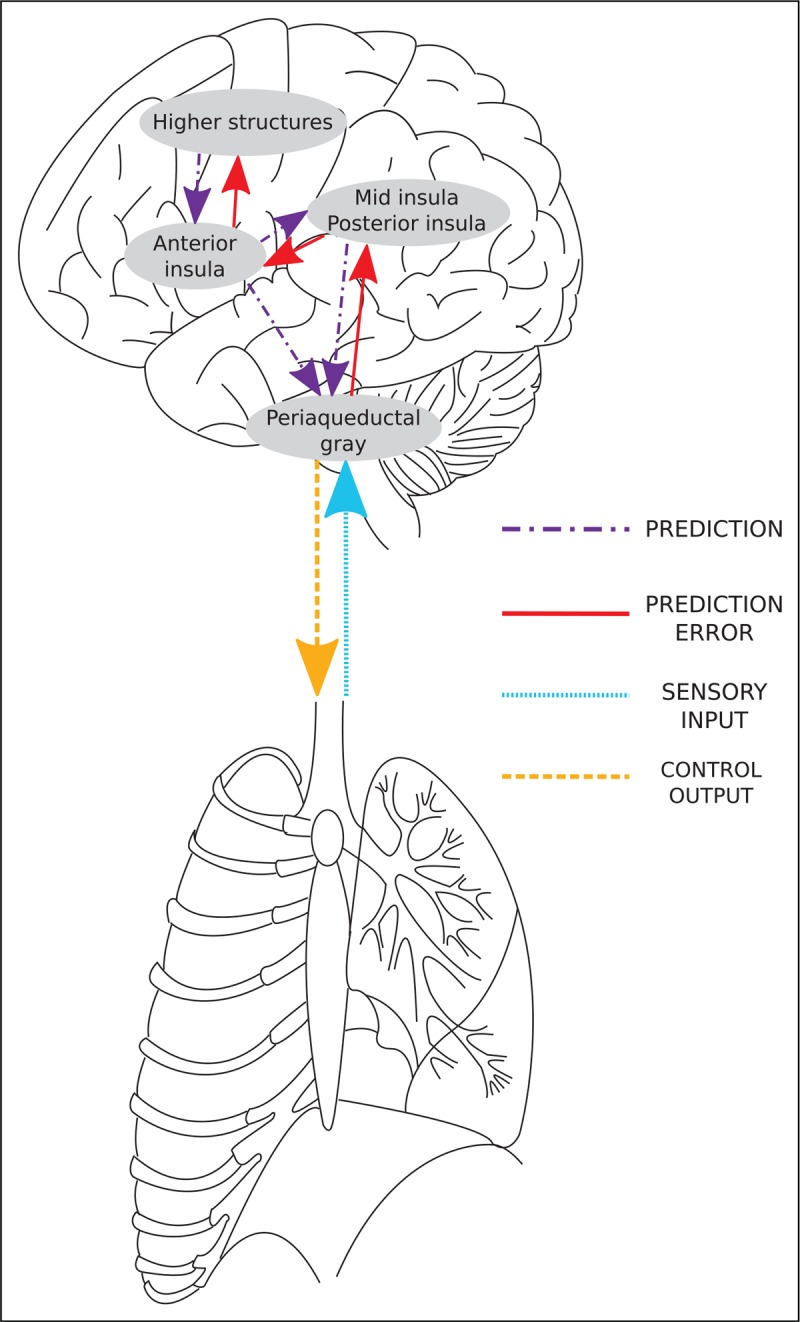
Potential neural network for breathlessness. Sensory signals arise in the body and are transmitted to the brain. Simultaneously, the brain generates predictions about the sensations the body should be feeling. When comparison between predictions and sensory information shows a mismatch, prediction error is generated. This error is transmitted back through the system, in a feedback loop, and prediction error is reduced by updating the brain's predictions or via action generation that alters incoming sensory information. Here we show a potential brain network structure that could carry out this process based on the network outlined by Stephan and colleagues [12]. The key brain areas include, but are not restricted to, the anterior insula, posterior insula, mid insula; higher brain structures including the anterior cingulate cortex and the orbitofrontal cortex; and brain stem nuclei and midbrain structures, such as the periaqueductal gray (PAG).

Current theories of interoception, including the Embodied Prediction Interoceptive Coding model (EPIC) [28] and the Neurovisceral Integration Model [29], have made suggestions regarding where predictions and prediction errors are generated [28], although evidence is currently scarce. These models hypothesize that predictions are generated in agranular, limbic cortices (such as the anterior cingulate cortex and anterior insula cortex), before being fed forward through a hierarchical network and compared with incoming sensory signals, leading to the generation of prediction errors at multiple levels (Fig. 2). The dynamic comparison and integration of these two signals leads to interoceptive perceptions, such as breathlessness. Furthermore, to reduce errors, prediction error magnitudes are thought to either drive learning via updating of predictions, or the generation of actions that alter incoming sensations to better match predictions [23]. Therefore, anything influencing the generation of predictions or their dynamic comparison with sensory afferents may alter breathlessness perception in combination with the peripheral environment.

## INFLUENCES ON BREATHLESSNESS PERCEPTION

Although the influence of expectations and affect (or more broadly, our emotions) on perceptions such as breathlessness have long been recognized [30–34], the mechanisms of action are currently unknown. Here, we explain our current understanding of the brain's involvement in breathlessness, and how factors such as expectation and affect can influence this perceptual system.

### Learned expectations

Expectations are known to have a significant influence on breathlessness. For example, in people with asthma, breathlessness-related cues (such as wheezing sounds) can induce breathlessness [31,32] and even measurable levels of bronchoconstriction [33]. Equally, simply observing breathlessness in others can induce mild-to-moderate breathlessness [35^▪^]. Furthermore, perceptions of breathlessness are increased when an individual returns to a context or situation in which breathlessness has been previously experienced, even in the absence of the original breathlessness stimulus [34,36]. As such, an individual with chronic breathlessness approaching a flight of stairs may feel breathless before even beginning to climb the stairs. The brain's predictions based on past experiences of stairs leading to breathlessness, and the expectations regarding similar situations thus motivate the perception.

Studies using functional neuroimaging have begun to highlight the brain areas associated with simple expectations of breathlessness. Conditioned anticipation of breathlessness [15–18,37] and breathlessness-related word cues [38–40] are associated with activations in a broad network including the anterior insula, anterior cingulate, prefrontal cortex, plus midbrain structures including the ventrolateral periaqueductal gray (PAG) [41^▪▪^,^42^,^43^]. Faull *et al.*[37] have uncovered initial evidence of dynamic actions within this network, whereby anticipatory brain activity may influence resulting perceptions. In this study, the strength of the disconnect between the ventrolateral PAG and both lateral PAG and motor cortices during anticipation reflected subsequent breathlessness perception. This work evidences the importance of expectations in breathlessness perception and has highlighted some of the brain areas involved. However, further investigations are required to elucidate the underlying brain mechanisms that link expectation and breathlessness.

### Affect

Affect has a demonstrable impact on breathlessness [44]. Depression and anxiety are major comorbidities in respiratory disease, cardiac disease and cancer [45–49]. Depression and anxiety are associated with increased breathlessness [50,51], increased mortality (depression is associated with almost doubled mortality over 1 year [30]) [52,53], a 10% increase in hospitalizations and diminished social and physical functioning (anxiety and depression are associated with a 13.7 point difference on the impact scale of the St George's Respiratory Questionnaire, on which 4 points is the minimally clinically important difference [54]) [23]. Furthermore, neuroimaging studies of breathlessness perception have identified activity in brain areas regularly associated with emotion and affective processing including the anterior cingulate cortex, insula and amygdala, as well as sensory processing areas [15,16,37,55–59]. In healthy participants, anxiety sensitivity (defined as anxiety towards bodily sensations) appears to be related to individual variability in breathlessness perceptions, and also to brain activity in the precuneus during both the anticipation and perception of breathlessness, the anterior insula during mild breathlessness and parietal sensorimotor areas during strong breathlessness [60]. Furthermore, induced negative affect during resistive loaded breathing gives rise to higher unpleasantness of perceived breathlessness relative to induced positive affect, and is associated with activity in the right anterior insula and the right amygdala [61]. Playing a role in both the anticipation and experience of breathlessness, affect, therefore, influences activity across a broad network of brain areas and has significant clinical implications.

Previous research has reported reduced breathlessness and negative emotionality following pulmonary rehabilitation [62], and there is evidence to suggest that breathlessness dimensions such as mastery are also improved [7]. Although changes in breathlessness-related anxiety have been associated with changes in activity in the superior parietal lobe, primary motor cortex, premotor cortex, posterior cingulate cortex and angular gyrus [38], it is not yet known how the dynamics of the underlying brain network associated with breathlessness are altered. Hence, we now look to predictive processing models to help us better understand the possible underlying mechanisms.

## POTENTIAL MECHANISMS OF BREATHLESSNESS PERCEPTION

The previous sections have described current knowledge of how expectation and affect are associated with breathlessness, and the regions of the brain that correlate with these observations. This section builds upon these findings and introduces the new ideas of predictive processing to highlight potential underlying mechanisms of breathlessness perception. The two main areas of discussion are as follows:

(1) Dampened sensory sensitivity. Different people have different thresholds at which they notice a change in their breathing. We will explain why reduced sensitivity to respiratory stimuli may alter perceptions of breathlessness in multiple ways.
(2) Precision and prediction. We will explain factors underlying how and why the brain may assign differing weights to predictions and incoming sensory signals when generating breathlessness perceptions. The key concepts here are:
  (a) Balance between predictions and incoming signals
  (b) Increased sensitivity to contextual cues (e.g. a flight of stairs or flowers)
  (c) Resilience of learned expectations to change in the face of contradictory evidence

### Dampened sensory sensitivity

Although dampened sensory sensitivity may typically lead to reduced perceptions of bodily sensations, perceptual accuracy may also be affected. For example, despite anxiety often being associated with greater vigilance towards threats and bodily sensations, it has been noted that anxiety may actually be related to dampened sensitivity to resistive respiratory stimuli [63]. Dampened sensitivity has also been observed in people with asthma [64]. This reduced sensitivity to respiratory events requires a greater change in stimulus to reach interoceptive thresholds, which can lead to greater reliance on expectations to help form perceptions. If paired with heightened vigilance, the result may exacerbate the dissociation between objective physiology and symptoms.

### Precision and prediction

Within a Bayesian predictive model of the brain, when sensory signals and predictions interact, it is the confidence or ‘precision’ values assigned to these inputs that play a key role in how the perception is generated [65]. The greater the precision assigned to a prediction or sensory input, the greater the reliance placed on that signal and the greater influence it has on the final perception of breathlessness. Precise predictions and imprecise sensory signals can cause perceptions to differ from the actual stimuli in the world. A simple example of this is the placebo effect, where expectation is directly modulated by an otherwise inert treatment [66^▪▪^–68^▪▪^].

The accuracy by which an individual can consciously monitor their own internal bodily signals (interoceptive ability) is a result of both the noise associated with incoming sensory information and the precision of concurrent predictions. This ability varies across individuals dependent on previous experiences [69,70]. We will now outline some of the potential avenues by which interoceptive abilities may be altered.

### The balance between predictions and incoming signals

**FIGURE 3:**
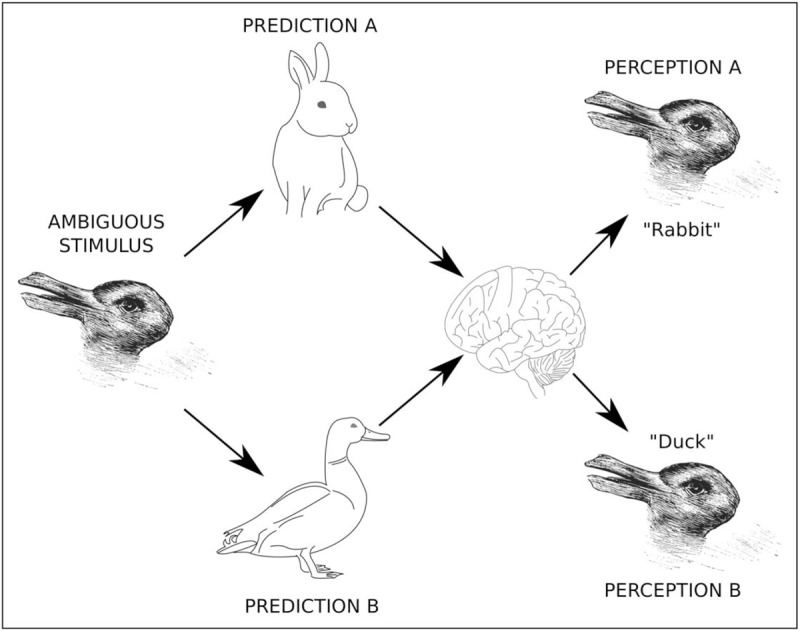
Influence of predictions on ambiguous stimuli. When a stimulus in the world is ambiguous, such as the classic rabbit-duck illusion, the brain's predictions have a significant influence on the final perception. When primed with the concept of a ‘rabbit’, the final perception is more likely to be that of a rabbit and vice versa for predictions of ‘duck’. In the context, of breathlessness, the sensations in the body, from those associated with low oxygen saturation to chest tightness and shortness of breath, can be ambiguous or of low intensity. When these sensations are ambiguous, the final breathlessness perception is far more dependent on brain's predictions. ‘Duck-rabbit illusion’ source: https://commons.wikimedia.org/wiki/File:Kaninchen_und_Ente.svg; https://digi.ub.uni-heidelberg.de/diglit/fb97/0147/image.

Within the Bayesian model, ambiguous stimuli produce imprecise incoming sensory signals, and thus perception is more dependent on the brain's predictions. An example taken from the visual system is shown in Fig. 3. Furthermore, increased symptom reporting in individuals with high negative emotionality [71,72] is particularly apparent when physical changes, such as airway constriction, are ambiguous or of low intensity [73]. Although negative emotionality appears to firstly bias predictions towards the presence of breathlessness, this influence would be amplified when combined with imprecise interoceptive sensory signals occurring at lower stimulus intensities. For example, when anxious, a vague, low-level respiratory sensation that may normally go unnoticed or be dismissed can be amplified, giving rise to the perception of breathlessness. When predictions bias perceptions away from true sensory signals, the sensory signals must be more extreme to pull perceptions back towards true sensation. Improving both bias and the precision of sensory signals would, therefore, increase the influence of sensation on perception, and reduce the impact of inaccurate predictions.

### Increased sensitivity to contextual cues

High symptom perceivers also appear to be more susceptible to contextual cues, such as the explicit suggestion of the presence of bronchoconstrictors or bronchodilators in the environment, demonstrating a reduced reliance on incoming sensory information [74]. Negative affect appears to magnify the influence of contextual cues on perception, such as when placebo inhalers improve breathlessness in asthma, and in particular in individuals susceptible to negative emotions [75]. Furthermore, negative contextual cues, such as foul smelling odours and explicit suggestion of unpleasant feelings, have more influence on breathlessness in susceptible individuals relative to positive cues [72,76]. It is possible that contextual cues may work to increase the precision of predictions in high symptom perceivers, and thus reduce the relative influence of sensory signals.

Although utilizing and learning environmental cues that accurately predict an outcome can be advantageous for maintaining our health, for inaccurate or false associations this aberrant learning is deleterious. In the context of breathlessness, reducing sensitivity to contextual cues (such as odours), explicit suggestion and stimuli relating to bronchoconstriction (such as flower pollen) could reduce the precision of the brain's predictions, and thus boost the relative influence of sensory signals on perceptions. This could allow a realignment of the sensory signal and breathlessness.

### Resilience of learned expectations

Several mechanisms may make perceptual expectations about breathlessness resilient to change. Active modulation (via changes in behaviour) of the interoceptive signal may mean individuals experience what they expect to experience [14^▪▪^] (the basis of commonly observed placebo effects). Additionally, biases in learning could influence prediction updating, meaning that incorrect predictions continue to have an influence despite their inaccuracy. Demonstrated in the pain literature, people tend to only accumulate and value the information that confirms their beliefs [14^▪▪^]. For example, if an individual expects to be breathless when they have forgotten their inhaler, the importance assigned to occasions of a forgotten inhaler where breathlessness ensued is likely to be greater than for equivalent occasions when the individual did not become breathless. This means that the expectation of breathlessness when without one's inhaler can persist even if the occasions on which breathlessness actually occurs are rare. Furthermore, learned expectations, based upon recalled breathlessness, can be biased by the difference between recalled and experienced breathlessness [77]. Such combined mechanisms allow for the persistence of inaccurate, although ‘precise’, predictions about breathing despite contrarian evidence.

In summary, within a Bayesian model of breathlessness, it is thought to be the balance between sensory signals and predictions that determines perception. Affect and expectations may influence this balance, and can drive perceptions to be based too heavily on predictions because of dampened sensory sensitivity and/or overly precise predictions. Increased sensitivity to contextual cues and the resilience of learned expectations can then easily lead to inaccurate, yet persistent predictions that drive perceptions of breathlessness away from objective measures of disease.

## LOOKING TO THE FUTURE

Considering breathlessness perception in terms of Bayesian theories of sensory processing, as exampled above, offers an informative new perspective [23,78,79]. Within the breathlessness literature, the previous focus on localized brain activity can now be updated to incorporate the influence of broad, dynamic brain networks.

Computational models offer one way to better understand the communications within these networks. This approach allows us to make inferences about the underlying mechanisms of breathlessness perception, which cannot be observed directly. A computational model mathematically formalizes the relationship between brain activity and other measures of interest (such as affect and expectations). A model's predictions should be testable via comparison with real data. The closer the model fits the real data, the more confident we can be that the model may be capturing some important aspects of the data. We can then test whether the model is capturing some important aspects of the data, and therefore potentially behaviour [80^▪^]. Existing neuroimaging work typically describes correlations between brain and behaviour, whereas computational modelling moves beyond correlations and allows us to generate and test hypotheses regarding the possible underlying mechanisms of breathlessness originating in the brain. For example, computational modelling could help elucidate how different columns of the periaqueductal gray communicate with the insula and sensory cortices to generate, update and/or compare predictions relating to breathlessness. This may allow us to demark the influences of predictions versus sensory interoceptive signals in the brain. Using these models to identify brain-based markers of inaccurate perceptions should then help us to understand reinforcement of maladaptive predictions and mechanisms of action for affect and emotion. Importantly, utilizing computational models could inform how expectations and affect influence breathlessness perception, leading us toward implementations for positive change for each individual.

## HELPING PATIENTS

Despite clinical observations, treatment of comorbid mood disorders in clinically breathless groups via anxiolytics or antidepressants does not produce the expected improvements in breathlessness [81,82^▪▪^]. These findings are not entirely surprising, as within the predictive processing framework, improving breathlessness is not simply about improving mood but also realigning the whole system of expectations, predictions and sensory processing.

Identifying specific neurocognitive markers related to over-weighted predictions, dampened sensory sensitivity and/or heightened effects of contextual cues should aid patient group stratification and tailored treatment plan development. By attempting to formally model breathlessness perception in the brain, we may expose neural targets for both pharmacological and nonpharmacological interventions, such as neurofeedback strategies, which focus on training specific brain pathways. Furthermore, predictive models could give rise to more accurate bedside measures that are developed from neural mechanisms, as well as predict successful drug and behavioural therapy combinations to be used in personalized treatment plans.

## CONCLUSION

Utilizing predictive models of symptom generation opens avenues of opportunity to progress our understanding of breathlessness. These advances could change the way that we measure and treat breathlessness using brain-based markers and a personalized approach. Current work outlining the brain networks of breathlessness perception, alongside modern neuroscientific thinking, provides a springboard for understanding causal mechanisms that have the potential to change the way we treat breathlessness.

## Acknowledgements

The authors would like to thank Professor Havi Carel and Mr Gwynne Reddick for their input and guidance on the experience of breathlessness. We would also like to thank Dr Sarah Booth and Dr Matthew Maddocks for their feedback on a previous version of the manuscript.

### Financial support and sponsorship

This work was supported by the JABBS Foundation, the Dunhill Medical Trust, and the NIHR Biomedical Research Centre based at Oxford University Hospitals NHS Trust and The University of Oxford. This article is based upon work funded by a Medical Research Council Clinician Scientist Fellowship (grant number G0802826) awarded to K.T.S.P. O.K.F. is a Marie Skłodowska-Curie Fellow who is supported by the European Union's Horizon 2020 research and innovation programme, under the grant agreement No 793580.

### Conflicts of interest

K.T.S.P. has acted as a consultant for Nektar Therapeutics. The work for Nektar has no bearing on the contents of this manuscript. The remaining authors have no conflicts of interest.

## REFERENCES AND RECOMMENDED READING

Papers of particular interest, published within the annual period of review, have been highlighted as:

▪ of special interest
▪▪ of outstanding interest
